# The Efficacy of Collagen Barrier Membranes in Regenerating Vertical Bone Defects: A Clinical and Cone Beam Computed Tomography (CBCT) Assessment

**DOI:** 10.7759/cureus.72550

**Published:** 2024-10-28

**Authors:** Tsvetalina Gerova-Vatsova

**Affiliations:** 1 Department of Periodontology and Dental Implantology, Medical University of Varna, Varna, BGR

**Keywords:** collagen barrier membrane, guided tissue regeneration, infrabony defects, periodontology, vertical bone defect

## Abstract

Context

The underlying principle of guided tissue regeneration (GTR) lies in the use of barrier membranes. Their role is key to this method, as they inhibit the rapid growth of epithelial and connective tissue cells, thus isolating the infrabony defects (IBDs) and ensuring the regeneration of slower-growing periodontal structures.

The main disadvantages of resorbable membranes are related to their limited time of action and the need to use them in two layers, which increases the chance of a postoperative complication, i.e., the dehiscence of the barrier membrane. In cases where barrier membranes are used alone, there is a risk of soft tissue “collapse” into the IBDs and disruption of the blood clot zone. This is why they are more commonly used in combination with bone repair material. However, when relatively smaller periodontal ​​​​​​IBDs are present, barrier membranes can be used alone.

It is such IBDs that are included in the present study that are relatively narrow and not as deep. The technique of GTR, with the sole application of a resorbable collagen membrane, was used. Clinical and radiographic results were evaluated and analyzed at the earliest possible stage after the intervention, which was the sixth month. In this way, we demonstrated the remarkable regenerative capabilities at an extremely early stage of the increasingly neglected GTR technique with the sole application of a barrier membrane.

Aim

Investigation into the efficacy of GTR for vertical IBDs utilizing solely applied barrier membranes assessed six months post-surgery.

Material and methods

The research was carried out from August 2022 to July 2023 at the Medical University Varna, Varna, Bulgaria, specifically within the Faculty of Dental Medicine, utilizing the University Medical and Dental Center as its basis. The study encompasses 12 cases featuring two-wall, tri-wall, or a combination of the specified vertical IBDs.

Following Ramfjord’s treatment sequence, an up-to-date periodontal status was recorded at the re-evaluation stage after the hygiene phase, and a cone beam computed tomography (CBCT) examination was ordered in the areas with vertical IBDs. Three clinical (probing pocket depth, gingival margin level, and clinical attachment level) and three radiographic parameters (A, B, and C) were evaluated immediately before the future surgical intervention.

Six months after the GTR with the sole application of a barrier collagen membrane, the same parameters studied at an earlier stage were recorded on all patients.

Results

The clinical outcomes observed at six months post-GTR utilizing a barrier membrane in vertical IBDs indicated an average reduction in probing depth of 4.17 mm, an average apical migration of the gingival margin of 0.33 mm, and an average gain of clinical attachment level of 3.83 mm. Bone filling is evident on the CBCT, corroborated by the following measurements: (A) an average reduction of 1.68 mm, (B) an average reduction of 0.50 mm, and (C) an average reduction of 0.11 mm.

The study’s impressive results are largely due to the relatively small number of cases included, requiring further improvement to confirm the method’s effectiveness.

Conclusions

The study confirms the potential of the membrane technique, although the extent of the healing process is assessed at an extremely early stage. It can be safely concluded that it is not always necessary to place bone repair material under the barrier membrane to obtain good healing results.

## Introduction

Bone regenerative techniques have been widely adopted in orthopedic surgery, dental implantology, and periodontology [1]. Their main focus is bone preservation in the first place and then bone regeneration in cases of bone deficiency [2-4].

The foundational theoretical ideas of guided tissue regeneration (GTR) were established by Melcher in 1976, who emphasized the importance of eliminating undesirable cell types from healing locations to facilitate the formation of targeted tissues [5]. Since that time, barrier membranes have continued to demonstrate successful results due to their ability to stop epithelial and connective tissue cells from entering the infrabony defects (IBDs) and to allow the growth of slower-growing periodontal structures [6-9].

At the histological level, using this method after the surface wettability of the barrier membrane, adsorption of plasma proteins occurs in the area between it and the tissue interface. These proteins can attract specific growth factors and progenitor cells, which play a key role in tissue regeneration. In addition to all this, the surface wettability of the barrier membrane influences platelet activation, which is inevitably associated with their degranulation and the release of more growth factors [10,11].

Collagen membranes have become established in periodontal regeneration and particularly in the treatment of vertical IBDs as early as 25 years ago [12]. With its many biological properties, collagen has a wide application in medicine today. It has low immunogenicity, adsorbs and activates fibroblast cells, and has a hemostatic function [11,13]. Furthermore, there is evidence that collagen membranes promote fibroblast DNA synthesis and more pronounced adhesion of osteoblast cells to the barrier surface [14].

There is a correlation between the degree of cross-linking of collagen fibers and the rate of membrane resorption. The more pronounced the cross-linking, the more slowly the barrier membrane resorbs [12]. Today, one of the most essential requirements for barrier membranes is to provide area and stability for the blood clot. In this way, they will create and maintain space in close proximity to the tissues we wish to regenerate [5]. This means that the resorbable membranes must degrade for the period required to regenerate our desired tissues [12].

The objective of this study is to evaluate and analyze the clinical and radiographic results of a known but largely forgotten method: GTR using only a barrier membrane in vertical bone defects. Based on the vast majority of publications in the databases, mainly investigating the methods of GTR with barrier membrane and bone repair material with or without additional bioactive materials, and analyzing the impressive results of the present study, it can be concluded that the use of bone repair material in the treatment of vertical bone defects is not necessary at all costs and that the method with barrier membrane application alone is extremely reliable.

## Materials and methods

The research was carried out from August 2022 to July 2023 at the Medical University Varna, Varna, Bulgaria, specifically within the Faculty of Dental Medicine, utilizing the University Medical and Dental Center as its basis. The study encompasses 12 cases (seven males and five females) featuring bipartite, tripartite, or a combination of the specified vertical IBDs. Ethical approval (No. 118/23 June 2022) was issued from the Research Ethics Committee of the Medical University of Varna. All participants included in the study were between 35 and 53 years old, female and male, non-smokers, and had an established vertical bone defect indicated for regenerative therapy. The set criteria for participation in the study were aged between 18 and 65 years; written declaration by the patients that they were systemically healthy, with no established systemic diseases; signed informed consent for the forthcoming treatment; and last but not least, satisfactory personal oral hygiene. The population with an age range between 18 and 65 years is the most numerous, and the majority of these patients are self-aware, sufficiently cooperative, motivated, and actively involved in the treatment by maintaining strict personal oral hygiene. 

Exclusion criteria for patients from the study include poor oral hygiene, untreated periodontal diseases, horizontal bone loss, vertical bone defects, local and systemic conditions that interfere with recovery, unsupervised systemic diseases, malignant diseases, previous radiation treatment in the mouth, smoking, and bisphosphonate use.

The collagen membrane chosen in this study was Jason membrane (Straumann) from porcine pericardium, containing multiple collagen fibers with a high degree of cross-linking. There are also elastic fibers that are bonded to the collagen fibers, and this further increases the mechanical stability of the membrane. The manufacturer presents a resorption time of three to six months after surgery.

**Figure 1:**
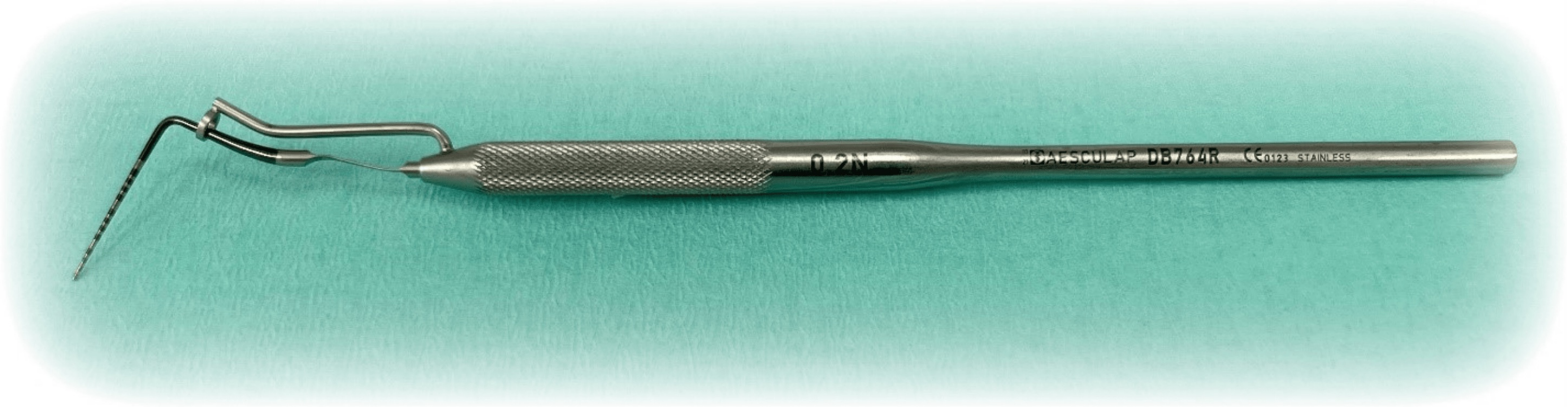
UNC 15 with standardized pressure

Following Ramfjord’s treatment sequence, an up-to-date periodontal status was recorded at the re-evaluation stage after the hygiene phase, and a cone beam computed tomography (CBCT) was ordered in the areas with vertical IBDs (at 0 months). Three clinical (probing pocket depth: the distance measured in mm from the gingival margin to the bottom of the pocket, gingival margin level: the distance measured in mm from the gingival margin to the cementoenamel junction (CEJ), and clinical attachment level: the distance in mm from the CEJ to the bottom of the pocket). The periodontal probe with which the measurements of the studied clinical parameters were performed was UNC 15 with standardized pressure (Figure 1).

CBCT was used to more accurately determine the size and contour of vertical IBDs. The CBCT score is a composite of parameters A (the distance from the CEJ to the bottom of the defect), parameter B (the distance from the CEJ to the bone crest of the defect), and parameter C (the distance of the widest range in the area of the bony defect). The listed clinical and radiographic parameters are assumed to be initial and measured immediately before the future surgical intervention. A Planmeca ProMax 3D cone beam tomography (3D scanner) was used for CBCT examination and image analysis.

Six months after the GTR with the sole application of a barrier collagen membrane, all patients showed the same parameters studied at an earlier stage. Statistical analysis of the data was performed (using analysis of variance (ANOVA), one-sample t-test, paired-samples t-test, and one-way ANOVA), and convincing results were presented regarding the effectiveness of this method at an extremely early stage after the treatment. 

Clinical protocol for GTR with the sole application of barrier membrane in vertical IBDs 

**Figure 2:**
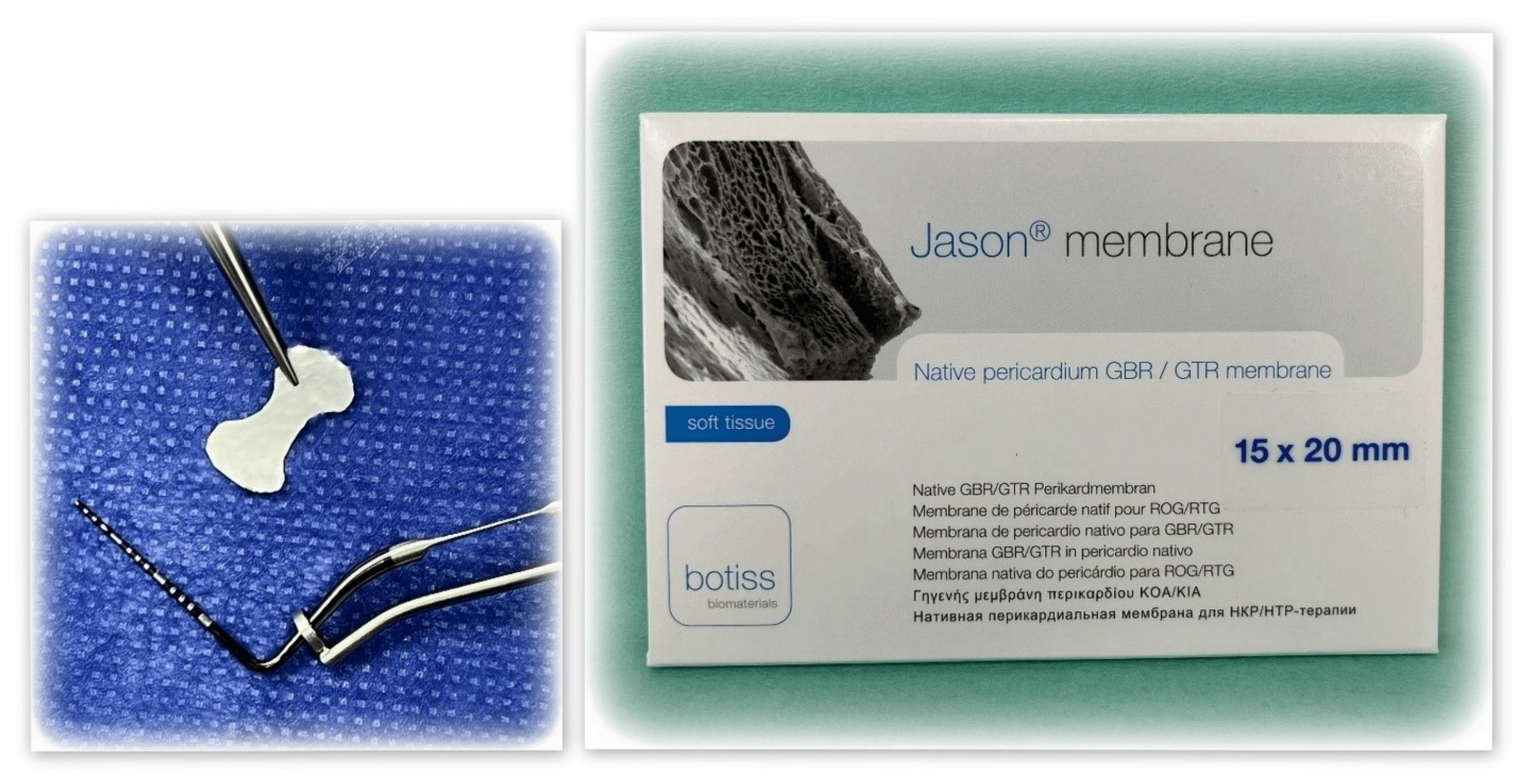
Image of botiss Jason membrane, Berlin, Germany

Local anesthesia is administered within the vertical bone defect using Septanest (Septodont, Saint-Maur-des-Fossés, France). An intrasulcular incision is made covering no more than two adjacent teeth medially and distally to the bone defect. Using an elevator, the mucoperiosteal flap is carefully debrided within a range that provides satisfactory visibility for the intervention. The defect is then cleaned of granulation tissue and flushed with saline. The root surface of the tooth with a vertical defect is treated using 24% EDTA gel (PrefGel, Straumann, Basel, Switzerland). This is followed by a precise flushing with saline. Finally, the vertical bone defect is closed using a surgical scissors-preformed pericardial collagen barrier membrane (botiss Jason membrane, Berlin, Germany) (Figure 2).

Once the collagen membrane is removed from the sterile packing, it is necessary to wet it with saline to make it more manipulatable and adaptable in the hands of the operator. The membrane should extend a minimum of 2 mm beyond the boundaries of the bone defect, and its edges should be adapted tightly to the bordering bone tissue and around the tooth necks to minimize the risk of epithelial and scar tissue ingrowth. Next, the flap is sutured with 5/0 Dafilon (B. Braun, Melsungen, Germany), taking care not to displace the membrane or perforate the suture material, which would compromise its function.

Postoperative care is standard. Patients within seven days are given antibiotics (Ospamox 1000 mg or Clindamycin 600 mg) and NSAIDs (Aulin 100 mg). For a period of 14 days, they are also prescribed an antibacterial mouthwash containing chlorhexidine digluconate (0.1%). At the end of the postoperative care period, a follow-up visit is planned by the patient to revise the surgical site and remove sutures.

## Results

Comparisons were made between the clinical and paraclinical indicators recorded 0 months before the patients’ surgical intervention and those measured six months following GTR with barrier membrane.

Probing pocket depth

**Figure 3:**
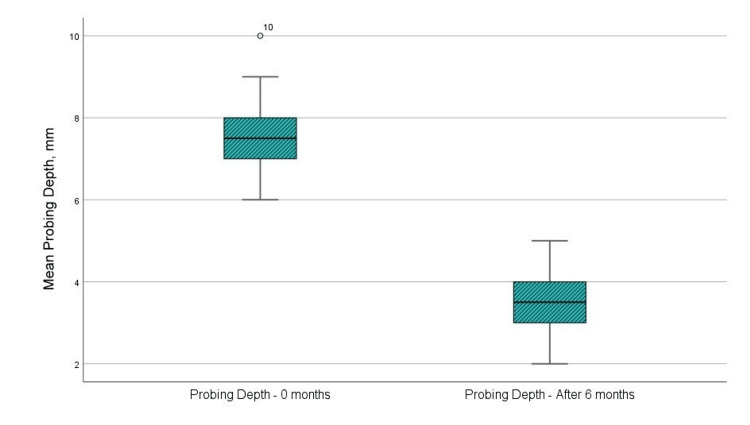
Mean values of the probing pocket depth at the examination before the surgical intervention and in the sixth month after the regenerative therapy

In the sixth month after directed tissue regeneration with a barrier membrane, the mean probing depth was 3.42 mm, compared to 7.58 mm before surgery (Figure 3 and Table 1). Statistical analysis using a t-test indicates a statistically significant variation in the “probing pocket depth” indicator (p < 0.001, p < 0.05, upper and lower significance limits not exceeding 0th, and t-value > 1.796, reference value at 11 degrees of freedom). The statistical results are presented in Table 2.

**Table 1:** Paired sample statistics with mean values and deviation CBCT, cone beam computed tomography

Paired sample statistics		Mean	N	Std. deviation	Std. error mean
Probing pocket depth	0 month	7.58	12	1.165	0.336
	6 months	3.42	12	0.9	0.26
Gingival margin level	0 month	-0.33	12	1.073	0.31
	6 months	-0.67	12	1.231	0.355
Clinical attachment level	0 month	-7.92	12	1.73	0.499
	6 months	-4.08	12	1.505	0.434
CBCT parameter А	0 month	7.14	12	1.031	0.298
	6 months	5.46	12	1.394	0.403
CBCT parameter В	0 month	3.61	12	0.988	0.285
	6 months	3.12	12	0.839	0.242
CBCT parameter С	0 month	1.89	12	0.408	0.118
	6 months	1.78	12	0.35	0.101

**Table 2:** Mean comparison and significance test CBCT, cone beam computed tomography

Paired sample test	Paired differences			95% confidence interval of the difference		Significance			
	Mean	Std. deviation	Std. error mean	Lower	Upper	t	df	One-sided p	Two-sided p
Probing pocket depth	4.167	0.835	0.241	3.636	4.697	17.289	11	<0.001	<0.001
Gingival margin level	0.333	0.778	0.225	-0.161	0.828	1.483	11	0.083	0.166
Clinical attachment level	-3.833	0.835	0.241	-4.364	-3.303	-15.906	11	<0.001	<0.001
CBCT parameter A	1.675	0.915	0.264	1.093	2.257	6.339	11	<0.001	<0.001
CBCT parameter B	0.495	0.354	0.102	0.27	0.72	4.839	11	<0.001	<0.001
CBCT parameter C	0.108	0.094	0.027	0.049	0.168	4.005	11	0.001	0.002

Gingival margin level

**Figure 4:**
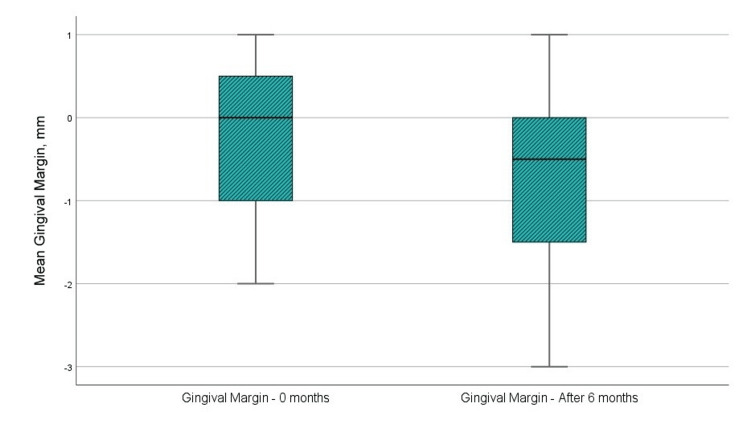
Mean values of the level of gingival margin at the examination before the surgical intervention and in the sixth month after the regenerative therapy

Figure 4 and Table 1 indicate that the mean level of gingival margin prior to surgical intervention was −0.33 mm, whereas the mean level at six months post-GTR with a barrier membrane was −0.67 mm. The t-test analysis indicated that the difference in “gingival margin level” was not statistically significant, with a p-value of 0.083, exceeding the 0.05 threshold. Additionally, the upper and lower significance limits are lower and higher than 0, and the t-value is below the reference value of 1.796 at 11 degrees of freedom. The statistical results are presented in Table 2.

Clinical attachment level

**Figure 5:**
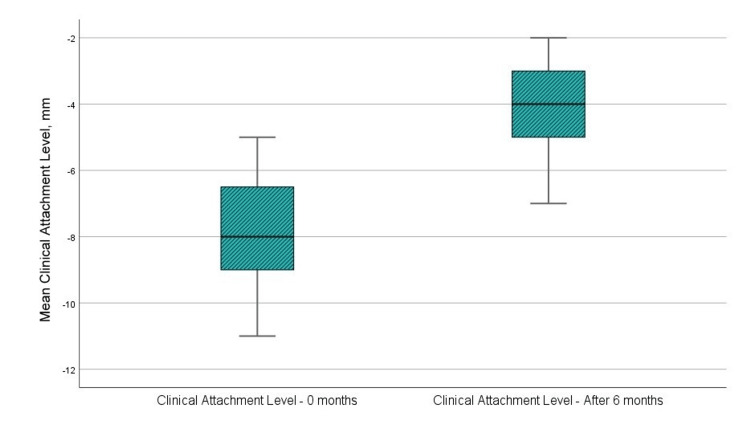
Mean values of the clinical attachment level at the examination before the surgical intervention and in the sixth month after the regenerative therapy

Figure 5 and Table 1 show that the mean clinical attachment level before surgery was 7.92 mm, whereas the mean level at sixth months post-GTR with a barrier membrane was 4.08 mm. The t-test analysis revealed a statistically significant variation in the “clinical attachment level” parameter (p < 0.001, p < 0.05, upper and lower significance limits < 0, and t-value > 1.796, the reference value at 11 degrees of freedom). The statistical results are presented in Table 2.

(A) Distance from CEJ to the bottom of the IBDs (by CBCT)

**Figure 6:**
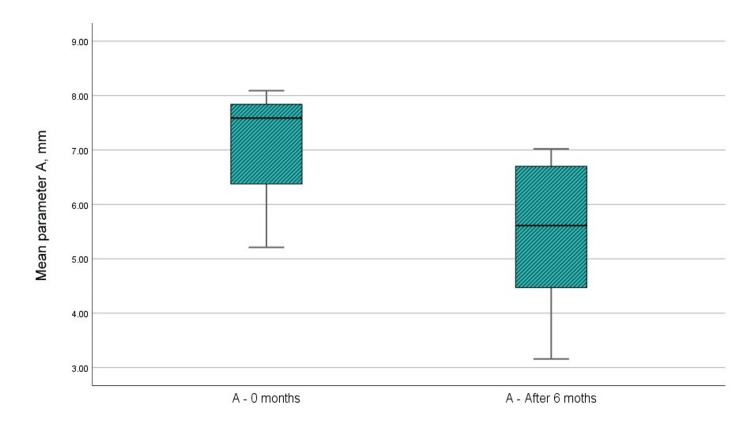
Mean values of the A parameter at the examination before the surgical intervention and in the sixth month after the regenerative therapy

Figure 6 and Table 1 indicate that the mean value of the paraclinical index “A” prior to surgical intervention was 7.14 mm, whereas the mean value at six months post-GTR with a barrier membrane was 5.46 mm. The t-test analysis indicated a statistically significant difference in the “A” parameter, with p < 0.001, which is below the 0.05 threshold. The upper and lower significance limits did not cross 0, and the t-value exceeded 1.796, the reference value at 11 degrees of freedom. The statistical results are presented in Table 2.

(B) Distance from CEJ to the crest of the IBDs (by CBCT)

**Figure 7:**
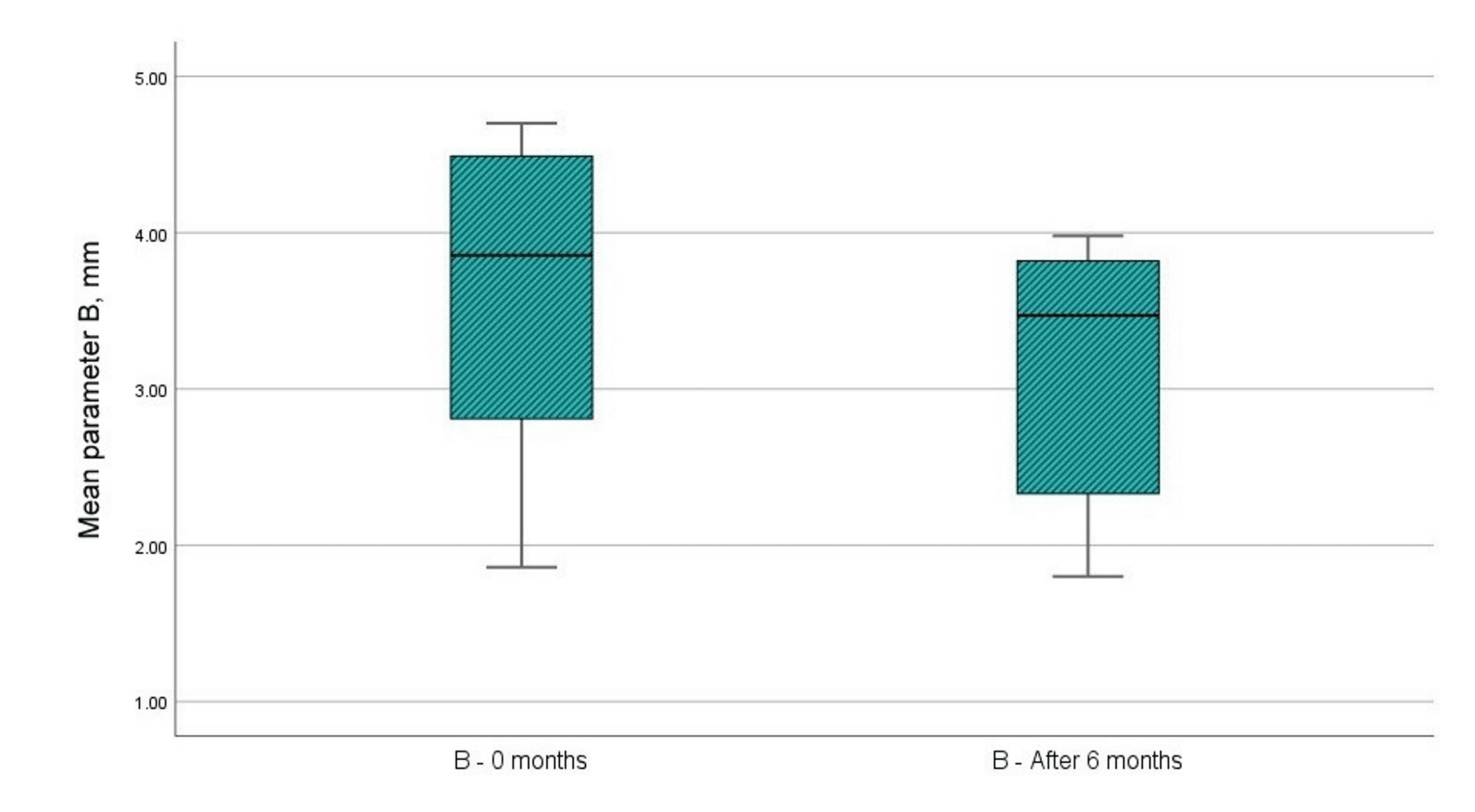
Mean values of the B parameter at the examination before the surgical intervention and in the sixth month after the regenerative therapy

Figure 7 and Table 1 indicate that the mean value of the paraclinical index “B” prior to the surgical intervention was 3.61 mm, whereas the mean value at six months post-GTR with a barrier membrane was 3.12 mm. Analysis using the t-test indicated that the difference in the “B” parameter was statistically significant, with p < 0.001, which is below the 0.05 threshold. The upper and lower limits of significance did not cross 0, and the t-value exceeded 1.796, the reference value at 11 degrees of freedom. The statistical results are presented in Table 2.

(C) Width of the IBDs (by CBCT)

**Figure 8:**
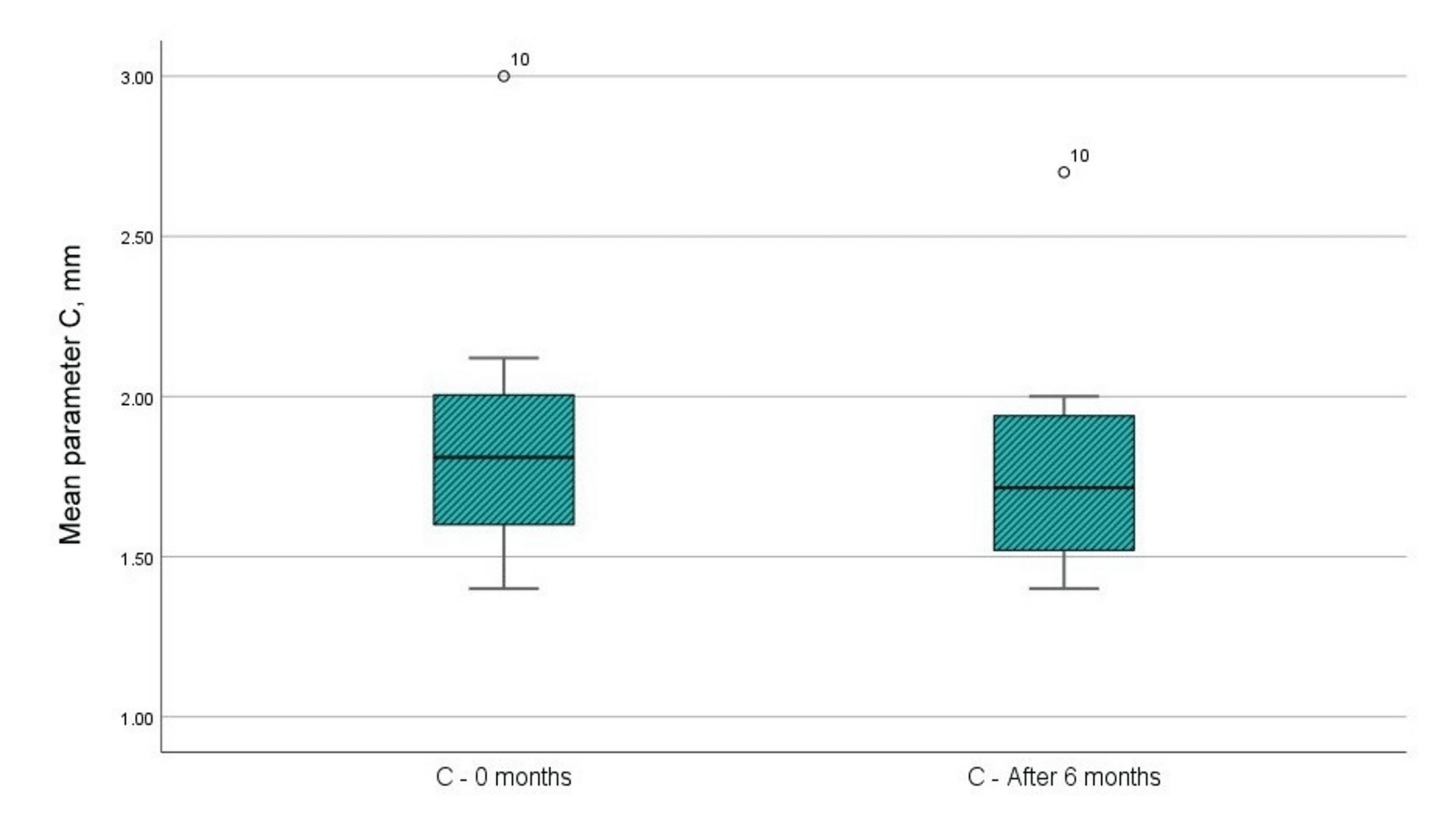
Mean values of the C parameter at the examination before the surgical intervention and in the sixth month after the regenerative therapy

Figure 8 and Table 1 indicate that the mean value of the paraclinical index “C” prior to the surgical intervention was 1.89 mm, whereas the mean value at six months post-GTR with a barrier membrane was 1.78 mm. The t-test analysis indicates a statistically significant difference in the “C” parameter, with p < 0.001, which is below the 0.05 threshold. The upper and lower significance limits do not cross 0, and the t-value exceeds the reference value of 1.796 at 11 degrees of freedom. The statistical results are presented in Table 2.

## Discussion

Many different barrier membranes are commercially available at present, and procedures are being initiated every day with the aim of researching and testing a new one that meets all the requirements [15]. The main characteristics that a barrier membrane should possess are biocompatibility, function to shape and maintain the defect space, isolation of rapidly growing cells and enabling regeneration of slower growing ones, and immunotolerance to the organism [16-18]. The effects of their applications can be investigated through radiological examination (CBCT) and histomorphometric assessment. The methods are the gold standard in alveolar bone evaluation [19,20].

Resorbable collagen membranes have a number of advantages over non-resorbable ones. First of all, they instantly fuse with the mucoperiosteal flap, and no additional fixation is needed. Also, due to the fact that they are resorbed by the enzymatic activity of macrophages and polymorphonuclear leukocytes, in cases where resorbable membranes are used, no second surgical intervention is necessary [7,12,21].

The main drawbacks of resorbable membranes are related to their limited time of action and the need to use them in two layers, which increases the chance of a postoperative complication, i.e., barrier membrane dehiscence. In cases where resorbable membranes are used alone, there is a risk of soft tissue “collapse” into the IBDs and disruption of the blood clot zone. They are, therefore, more commonly used in combination with bone repair material [6,7,22-24]. However, when relatively smaller periodontal IBDs are present, it is also possible to use resorbable barrier membranes alone.

Nowadays, it is easy to find information in the literature regarding the fact that collagen membranes lead to uniform results comparable to those of non-resorbable membranes in GTR [24,25]. For example, in 2017, Sheikh et al. [12] extensively reviewed the available literature related to the use and effectiveness of resorbable collagen membranes in periodontal IBDs. The team found enviable efficacy potential and comparable clinical and radiological outcomes to those of non-resorbable membranes.

The use of collagen membranes in the treatment of vertical IBDs, as discussed above, dates back decades, but in a vast body of literature, they are used in combination with a different type of bone repair material [26-29]. Analysis of the data from the present study presents the previously underestimated clinical potential of solely applied barrier membranes and finds statistically significant modification of the IBDs within a six-month period after GTR.

The limitations of the study are related to the fact that it was conducted over a short period of time (only one year) and that the follow-up time for the results was too short (six months after the surgical intervention). Another limitation that can be pointed out is that the present study was conducted in only one institution and included a relatively small number of patients. Last but not least, the limitations related to the eligibility criteria in the study should be noted, mainly considering the age range, patients who do not smoke, and the level of personal oral hygiene.

Despite the named limitations of our study, it should be noted that to date there is no publication investigating CBCT results after application of this method. CBCT is of utmost importance in these cases, as it is the only way to establish the dimensions and exact contours of periodontal IBDs.

## Conclusions

Statistical analysis of the data from the present study presented convincing results regarding the effectiveness of GTR with self-administration of a barrier collagen membrane in the treatment of vertical IBDs. The study confirms the potential of the membrane technique, although the extent of the healing process is assessed at an extremely early stage. It can be safely concluded that it is not always necessary to place bone repair material under the barrier membrane to obtain good healing results. This is, of course, preliminary evidence due to the short follow-up period and a limited number of cases. Further studies are therefore needed in this context. 

Periodontal regenerative therapy is expected to continue evolving, but one thing is certain: barrier membranes will remain an indispensable part of this treatment.
